# MPH Capstone experiences: promising practices and lessons learned

**DOI:** 10.3389/fpubh.2023.1129330

**Published:** 2023-05-11

**Authors:** Meg Landfried, Elizabeth Chen, Lindsay Bau Savelli, Morgan Cooper, Brittany Nicole Price, Dane Emmerling

**Affiliations:** Department of Health Behavior, Gillings School of Global Public Health, The University of North Carolina at Chapel Hill, Chapel Hill, NC, United States

**Keywords:** Capstone, culminating experience, service-learning, community partner, graduate public health education, MPH, accreditation, integrative learning experience

## Abstract

To ensure workforce readiness, graduate-level public health training programs must prepare students to collaborate with communities on improving public health practice and tools. The Council on Education for Public Health (CEPH) requires Master of Public Health (MPH) students to complete an Integrative Learning Experience (ILE) at the end of their program of study that yields a high-quality written product demonstrating synthesis of competencies. CEPH suggests written products ideally be “developed and delivered in a manner that is useful to external stakeholders, such as non-profit or governmental organizations.” However, there are limited examples of the ILE pedagogies and practices most likely to yield mutual benefit for students and community partners. To address this gap, we describe a community-led, year-long, group-based ILE for MPH students, called Capstone. This service-learning course aims to (1) increase capacity of students and partner organizations to address public health issues and promote health equity; (2) create new or improved public health resources, programs, services, and policies that promote health equity; (3) enhance student preparedness and marketability for careers in public health; and (4) strengthen campus-community partnerships. Since 2009, 127 Capstone teams affiliated with the Department of Health Behavior at the Gillings School of Global Public Health at The University of North Carolina at Chapel Hill have worked with seventy-nine partner organizations to provide over 103,000 h of in-kind service and produce 635 unique products or “deliverables.” This paper describes key promising practices of Capstone, specifically its staffing model; approach to project recruitment, selection, and matching; course format; and assignments. Using course evaluation data, we summarize student and community partner outcomes. Next, we share lessons learned from 13 years of program implementation and future directions for continuing to maximize student and community partner benefits. Finally, we provide recommendations for other programs interested in replicating the Capstone model.

## Introduction

Responding to public health crises like the COVID-19 pandemic requires a public health workforce skilled in community partnership (1, 2). Schools and programs of public health are thus charged with designing community-engaged learning experiences while also satisfying accreditation criteria (3). The accrediting body for schools and programs of public health, the Council on Education for Public Health (CEPH), requires Master of Public Health (MPH) students to complete an Integrative Learning Experience (ILE), which represents a culminating experience near the end of their program of study. The ILE must yield a high-quality written product (e.g., “program evaluation report, training manual, policy statement, take-home comprehensive essay exam, legislative testimony with accompanying supporting research, etc.”) that demonstrates synthesis of a set of competencies (2). Such products may be generated from practice-based projects, essay-based comprehensive exams, capstone programs, or integrative seminars (2). CEPH guidelines suggest ILE written products ideally be “developed and delivered in a manner that is useful to external stakeholders, such as non-profit or governmental organizations” (2).

Within this paper, we describe promising practices employed within a community-led, group-based, year-long, critical service-learning course, called Capstone, for MPH students within the Department of Health Behavior at the Gillings School of Global Public Health (Gillings) at The University of North Carolina at Chapel Hill (UNC-CH) (4). We explain the specifics of Capstone's staffing model; project recruitment, selection, and matching processes; course format; and assignments, all of which are designed to promote mutual benefit for students and community partners. Using internal and school-level course evaluations, we present findings on student and community partner outcomes. Next, we reflect on lessons learned from 13 years of implementation experience and suggest future directions for Capstone programming. Finally, we share recommendations for other programs interested in replicating Capstone. We hope the information presented in this paper will benefit other programs interested in ILEs that have mutual benefit for students and community partners.

## Pedagogical framework

By design, Capstone is a critical service-learning course. Service-learning pedagogies and practices vary widely. Essential elements of service-learning include community-engaged activities tied to learning goals and ongoing reflection (5–7). The literature documents wide-ranging benefits students gain from service-learning programs such as improved critical thinking skills as well as stronger leadership, communication, and interpersonal skills (5, 8). Participation in service-learning courses promotes program satisfaction (9), academic achievement (5, 8–10), and job marketability (9, 11) among students. Finally, service-learning experiences enhance students' civic engagement (2, 4, 7), cultural awareness, and practice of cultural humility (8, 12).

Despite these benefits, service-learning implementation challenges are well documented. Service-learning courses require significant resources to cover program expenses and staffing dedicated to developing and maintaining community partner relationships (7, 12–15). In addition, the academic calendar may not align with community partners' timelines (5, 14, 16). Students and community partners have additional responsibilities and competing priorities outside coursework, thus creating variable levels of engagement across program participants (13–15, 17, 18). In cases where students have nascent project management skills and limited professional experience (9, 10, 13), it can be difficult to achieve mutual benefits among students and community partners.

A prominent debate within the field is the degree to which service-learning projects perpetuate the status quo or facilitate social change. Specifically, researchers question which elements of service-learning best create the conditions for student learning and positive community transformation (5, 19–21). To provide a framework for this debate, Mitchell (5) differentiates between “traditional service-learning” and “critical service-learning.” Traditional service-learning is often critiqued for prioritizing student learning needs over benefits to the community (5, 21). In contrast, critical service-learning is explicitly committed to social justice (5). Key elements of a critical service-learning approach include: (1) redistributing power among members of the partnership; (2) building authentic relationships (i.e., those characterized by connection, mutual benefits, prolonged engagement, trust, and solidarity); and (3) working from a social change perspective (5).

Most service-learning program descriptions within public health training do not reference either a traditional or critical service-learning framework (8, 9, 11, 13, 14, 22, 23). Several published programs align with a traditional service-learning model, due to the exclusive focus on student benefits and the absence of an explicit commitment to power sharing, authentic partnerships, or social change. For example, Schober et al. (24) underscore service-learning as an effective means to train a younger workforce to address complex public health issues. Gupta et al. (8) describe the importance of self-reflection activities for personal growth and skill development, structured within a service-learning program for undergraduate students enrolled in a community nutrition course. While these courses contain many of the best practices in service-learning, including reflection, they discuss student outcomes without promoting or evaluating social change (6).

The literature also cites programs and courses that include elements of critical service-learning but do not use critical service-learning terminology. For example, a service-learning program at the University of Connecticut outlines how students contribute to structural changes and social progress through policy development and implementation as part of their applied practice experience, which culminates with a presentation to the state legislature (23). Additionally, Sabo et al. (12) describe a service-learning course at the University of Arizona oriented toward social justice, as the course is “modeled on the reduction of health disparities through exploration, reflection, and action on the social determinants of health” through strong community-academic partnerships across urban, rural, and indigenous settings. These examples highlight commitment to social progress, community impact, and equitable collaboration without overtly applying the language of critical service-learning.

A small number of service-learning practitioners define their programs explicitly as critical-service learning. Mackenzie et al. (13) document the benefits of a critical service-learning experience for undergraduate public health students, endorsing it as a “feasible, sustainable” high-impact practice. In their model, students partner with community organizations to address social determinants of health; analyze and challenge power dynamics and systems of oppression; and gain skills. As evidence of power sharing and social change, the authors document that communities have continued their partnerships with the university due to the expansive reach and impact of their collaborations. Authentic relationships were also developed as students gained a stronger sense of commitment to communities. Derreth and Wear (25) describe the transition to an online critical service-learning course as universities grappled with changing instructional formats with the onset of the COVID-19 pandemic. In this course, public health students collaborated with Baltimore residents to create evaluation tools while participating in reflective activities. As evidence of critical service-learning, they documented students' changed perspectives, ongoing commitment to collaborate with residents after the course, and development of strong connections with faculty. These courses show the possibilities of critical service-learning ILEs. Detailed descriptions of program structures are needed for interested faculty to replicate best practices. To assist others with adopting or adapting elements of critical service-learning ILEs, this paper provides specifics about Capstone programming.

## Learning environment

### Program overview

Community-Led Capstone Project: Part I and II (Capstone) is a graduate-level course situated within UNC-CH's Gillings' Department of Health Behavior (Department). The Department developed Capstone in response to faculty concerns about the variable investment in and quality of master's papers (26), coupled with a desire to design a practice-based culminating experience driven by community partners' needs, interests, and concerns. Capstone satisfies CEPH ILE requirements and serves as the substitute for UNC-CH's master's thesis requirement for students in the Health Behavior (HB) and Health Equity, Social Justice, and Human Rights (EQUITY) MPH concentrations. The overwhelming majority of students in these two concentrations are full-time residential students pursuing an MPH within a two-year time frame, though there are a few students who are enrolled in a dual degree program to earn their MPH alongside a Master of Social Work (MSW) or Master of City and Regional Planning (MCRP) within 3 years.

**Figure 1:**
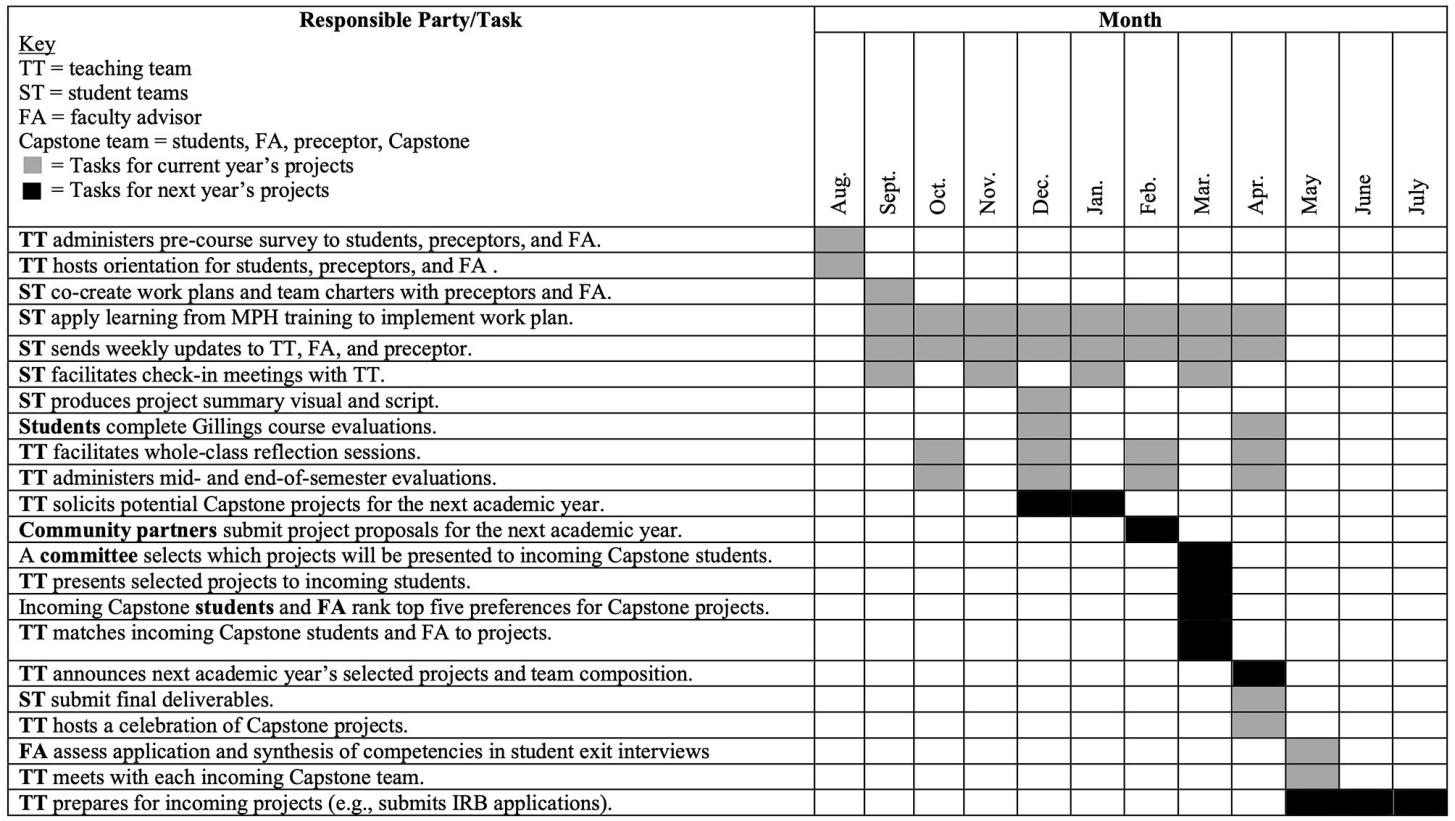
Gantt chart illustrating major Capstone activities and timeline.

During this year-long course, which occurs during the second year of the MPH program, students synthesize and apply their MPH training to community-designed public health projects. Supplementary material A, B include a list of HB and EQUITY required courses and their sequencing. The specific competencies applied and assessed during Capstone are listed in Supplementary material C. Each team of four to five Capstone students works with a partner organization and its constituents to produce a set of four to six deliverables (i.e., tangible products). Deliverables are based on the partner organization's self-identified needs. This community-led approach prioritizes partners' interests and gives students an opportunity to do applied public health work on a range of topics with a variety of organization types. Figure 1 details the tasks and timelines entailed in this programming. Table 1 presents information from selected projects that showcase the range of partner organizations, activities, and deliverables present in Capstone. Capstone's specific objectives are to (1) increase capacity among students and partner organizations to address public health issues and promote health equity; (2) create new or improved public health resources, programs, services, and policies that advance health equity; (3) enhance student preparedness and marketability for public health careers; and (4) strengthen campus-community partnerships.

**Table 1:** Sample projects.

**Partner organization**	**Project title**	**Deliverables**
Campus and Community Coalition to Reduce the Negative Impacts of High Risk Drinking, Chapel Hill Downtown Partnership (2018-2019)	*Measuring and Sharing the Efforts of the Campus and Community Coalition*	1. Data analysis report 2. Communication plan 3. Qualitative analysis report 4. Evaluation recommendations report
Chapel Hill-Carrboro City Schools (2016-2017)	*Needs Assessment for Community Mental Health Among Frank Porter Graham Bilingüe Staff and Parents in Chapel Hill, North Carolina*	1. Interview and Focus Group Guides 2. Formative Research Report 3. Community Resource Guide 4. Recommendations Report
Chatham County Council on Aging^*^ (2019-2020)	*Implementation, Evaluation, and Resource Development for Chatham County Council on Aging's Community Ambassador Program*	1. Community ambassador resources 2. Monitoring report 3. Evaluation toolkit 4. Communications workplan
El Pueblo, Inc.^*^ (2011-2012)	*Strengthening El Pueblo's Sexual and Reproductive Health Program for Latino/a Youth in North Carolina*	1. Funding guide 2. Community Assessment Report 3. Revised Curriculum 4. Strategic Guide
North Carolina Harm Reduction Coalition^*^ (2012-2013)	*Preventing Unintentional Drug Overdose in North Carolina by Advocating for Policies that Support Overdose Prevention*	1. Literature review summary fact sheet 2. Policy recommendations 3. Presentation 4. Legislative summit
Rural Opportunity Institute^*^ (2021-2022)	*Evaluating an Adaptation of the Social Accelerator Model for Rural Public Institutions Focused on Healing Trauma and Building Resilience*	1. Interview guides 2. Interview codebook and summary code report 3. Manuscript
Southern Coalition for Social Justice (2021-2022)	*Analyzing and Evaluating Strategies to Decriminalize Adolescence and Developing a Participatory Research Plan to Work with Youth Impacted by the Criminal Legal System*	1. Landscape analysis 2. Interview guide and transcripts 3. Program plan 4. Partner case studies and recommendations report 5. External report

* Capstone partner organization that has hosted multiple teams.

### Personnel and resources

Capstone involves numerous constituents and requires dedicated resources. Each partner organization is represented by one or two preceptors (i.e., main points of contact from the partner organization) who provide a vision for, direct, and supervise the project work. Preceptors spend 2–4 h per week meeting with students, providing guidance on the work, and reviewing deliverables. Student teams are responsible for managing Capstone relationships, processes, and tasks and producing deliverables that enhance their skillsets while meeting their partner organization's needs. They are expected to spend 6–9 h per week, outside of class time, on Capstone. One faculty adviser per project provides technical expertise and ensures that each team's project deliverables meet UNC-CH's master's thesis substitute and CEPH ILE requirements. Faculty advisers spend 30 min to an hour a week providing feedback and guidance on the project work. Advising a Capstone team every other year is a service expectation for Department faculty. The teaching team, which is comprised of course instructor(s) and teaching assistants (TAs), recruits the partner organizations and oversees and supports the Capstone experience. Each instructor manages ten to eleven teams (typically between forty and fifty students) and receives coverage equal to twenty percent full-time equivalent per semester. TAs, who are HB or EQUITY MPH alumni and/or HB doctoral students, each work with five to six teams and are expected to work 18 h a week on Capstone. TAs provide feedback on draft deliverables, direct students to resources, and help problem solve. Departmental administrative staff provide additional support to coordinate expenses associated with the program such as project-related travel, equipment, services (e.g., transcription, interpretation, translation), books, software, incentives, postage, and other costs. Capstone students pay a one-time $600 field fee to cover a portion of the expenses associated with Capstone. This fee was approved by the University and is paid when a student enrolls in the first semester of the course.

### Project recruitment, selection, and matching

#### Recruitment

The process of setting up Capstone projects takes 9 months of advance planning (see Figure 1). The Capstone teaching team solicits project proposals in December for the upcoming academic year. They send email solicitations with Capstone overview information (Supplementary material D) and the project proposal form (Supplementary material E) to current and former Capstone partner organizations, hosts of other experiential education experiences, and department listservs. The Capstone teaching team encourages recipients to share the solicitation information with their networks. Prospective partners' first step is to have an informational interview with a Capstone instructor to discuss their project ideas and to receive coaching on elements of successful proposals. These interviews are also an opportunity for the teaching team to assess an organization's capacity to support a student team and gain insights on the prospective preceptors' communication, work, and leadership styles. The teaching team invites prospective partners to submit draft proposals for their review prior to the proposal deadline. Prospective partners submit their finalized project proposals and a letter of support from their leadership to the teaching team by email in early February.

#### Selection

The teaching team typically receives twenty project proposals. To determine which projects will be presented to incoming Capstone students, a committee consisting of the teaching team and student representatives from the current Capstone class reviews and scores proposals based on the criteria listed in Table 2. Reviewers score each criterion on a scale of one through five with one being the lowest score and five being the highest score. The fifteen community partners with the highest scoring proposals are invited to share their ideas with students via a recorded seven-minute project overview presentation.

**Table 2:** Project selection criteria.

**Selection criteria**	**What to look for**
Project Scope	1. Is there a clear scope of work with tangible outputs that have clear purposes and steps, are interrelated, and connect to one overarching project goal? 2. Is the proposed scope of work appropriate and feasible for a team of students within the academic timeline? 3. Is there sufficient time and effort allocated to onboarding students to the project work and partner organization? 4. Will the project facilitate knowledge and skill acquisition and application that will enhance students' readiness for public health careers?
Organizational Capacity	1. Does the preceptor have demonstrated time, expertise, and interest to mentor public health students? 2. Does leadership at the partner organization demonstrate support for the project?
Equity	1. Does the partner organization demonstrate commitment to promoting health equity and social justice? 2. Were the people who will be most impacted by the project work involved in the project design? 3. Will students engage with the intended beneficiaries of the work?
Impact	1. Does the project have strong potential to make a meaningful difference in the health of the beneficiary communities and populations?

#### Matching

Incoming Capstone students have 1 week in March to review the proposal materials and rank their top five project preferences. Based on student rankings, the teaching team assembles project teams using the following guiding principles: (1) give as many students as possible their top-ranked project; (2) promote diversity of concentrations and experience levels within student teams; and (3) ensure the number of students per team is appropriate for the proposed scope of work. Once the student teams are assembled, the teaching team matches faculty advisers to projects based on faculty's interests and expertise. The teaching team announces final team composition in early April. The course instructor(s) facilitates an initial meeting with each student team, their preceptor(s), and their faculty adviser in May to build community, clarify expectations, and orient the student team to their project work and partner organization. Project work formally begins in August of the following academic year.

### Course format

Capstone spans the fall and spring semesters (fifteen weeks per term) and is three credits per term. To help students, preceptors, and faculty advisers become familiar with expectations for Capstone, the teaching team reserves the first 4 weeks of the fall semester for onboarding. As part of the onboarding process, each team cocreates a team charter (Supplementary material F) to promote authentic relationships between students and their community partners and to clarify expectations for working together. They also produce a workplan (Supplementary material G), which elaborates on the partner's project proposal, to outline the team's scope of work. After the onboarding weeks, the teaching team meets with each student team during class three times per semester to receive project updates and provide support. The teaching team facilitates two whole-class reflection sessions per semester to help students make meaning of their experiences. All other Capstone class sessions are protected time for student teams to meet and work on their projects.

### Course assignments

Capstone assignments are designed to ensure a mutually beneficial experience for students and community partners. They are also intended to facilitate critical reflection, yield high-quality written products, assess synthesis of selected competencies, and evaluate how students steward the relationships, processes, and tasks associated with their projects. To share power and collect their unique perspectives, preceptors and faculty advisers participate in the grading process. Tables 3, 4 summarize course assignments, their descriptions, whether they are completed and assessed at the individual or group level, and the party responsible for assessing the assignment.

**Table 3:** Capstone assignments for the fall semester.

**Assignment**	**Description**	**Assessment type**	**Assessed by**	**Percent of final course grade**
Pre-course survey	Qualtrics survey distributed by the teaching team to students, preceptors, and faculty advisers to create a shared understanding of the team members' expectations for the Capstone experience.	Individual	TT	0%
Weekly updates	Email sent by the student team using a template prescribed by the teaching team to create communication efficiencies and systematically keep the teaching team, preceptors, and faculty advisers updated on students' project work.	Group	TT	10%
Teaching team check-in meeting facilitation	Thirty-minute meeting facilitated by the student team to build community with, update, and receive support from the teaching team.	Group	TT	10%
Team charter	Microsoft Word document following a template (Supplementary material F) provided by the teaching team used to promote authentic relationships between Capstone students, their preceptor(s), and their faculty adviser by clarifying expectations for working together.	Group	TT	10%
Work plan	Microsoft Word document following a template (Supplementary material G) provided by the teaching team that clarifies the Capstone student team's scope of work by outlining the project deliverables, their steps, and their timeline.	Group	TT	10%
Project Summary Visual and Script	Power point slide and accompanying narrative text used to explain the team's project work and its intended impacts in preparation for being on the job market.	Group	TT	5%
Mid and End-of Semester Evaluations	Qualtrics surveys administered by the teaching team to students, preceptors, and faculty advisers to reflect on accomplishments and challenges and assess roles, responsibilities, processes, and deliverables.	Individual	TT	0%
Project management	Assessment of teams' management of Capstone project relationships, processes, and tasks.	Group	TT, P, FA	35%
Project participation	Assessment of individuals' contributions to the Capstone project.	Individual	TT, P, FA	20%

TT, Teaching Team; P, Preceptor; FA, Faculty Adviser.

**Table 4:** Capstone assignments for the spring semester.

**Assignment**	**Description**	**Assessment type**	**Assessed by**	**Percent of final course grade**
Weekly updates	Email sent by the student team using a template (Supplementary material D) prescribed by the teaching team to create communication efficiencies and systematically keep the teaching team, preceptors, and faculty advisers updated on students' project work.	Group	TT	10%
Teaching team check-in meeting facilitation	Thirty-minute meeting facilitated by the student team to build community with, update, and receive support from the teaching team.	Group	TT	10%
Mid and end of semester evaluations	Qualtrics surveys administered by the teaching team to students, preceptors, and faculty advisers to reflect on accomplishments and challenges and assess roles, responsibilities, processes, and deliverables.	Individual	TT	0%
Deliverables	Tangible products produced by the student team that are mutually beneficial to students' professional development goals and partner organizations' needs.	Group	TT, P, FA	35%
Project management	Assessment of teams' management of Capstone project relationships, processes, and tasks.	Group	TT, P, FA	20%
Project participation	Assessment of individuals' contributions to the Capstone project.	Individual	TT, P, FA	20%
Exit interview and prep sheet	Interview between student and faculty adviser to assess the student's synthesis and demonstration of foundational and concentration competencies.	Individual	FA	5%

TT, Teaching Team; P, Preceptor; FA, Faculty Adviser.

## Program evaluation

This study was exempted by UNC Chapel Hill's Institutional Review Board (IRB 21-0510) as it fell under the exemption category of “educational setting,” which includes research on instructional approaches and their effectiveness. To abstract and analyze data on the number of students who have completed Capstone, hours they dedicated to Capstone activities, and deliverables they produced, two authors referenced course records starting in 2009. The teaching team collects students' and preceptors' perspectives on Capstone through mid- and end-of-semester evaluations using Qualtrics. Gillings administers end-of-semester course evaluations that provide additional insights into student outcomes.

Core aspects of Capstone (e.g., program aims and our staffing model) have remained constant over the past 13 years. However, a variety of lessons learned and external conditions have led to program changes. Use of class time and project recruitment, selection, and matching processes have evolved to further promote health equity and maximize mutual student and community partner benefit. The EQUITY concentration joined Capstone in 2020, which led to changes in team composition. Furthermore, the COVID-19 pandemic necessitated a transition from in-person to a remote course format in academic years 2020 and 2021, introducing the opportunity to work with organizations across the nation.

To present qualitative findings that reflect our most current programming, two authors analyzed data from academic years 2020 and 2021. Ninety-eight students and twenty-two preceptors participated in Capstone during that time. The teaching team received a 100 percent response rate to their mid and end-of semester evaluations completed by students and preceptors and a seventy-two percent response rate to the Gillings-administered student course evaluations during academic years 2020 and 2021.

To identify key outcomes for students and preceptors, two authors completed a thematic analysis of evaluation responses (27, 28). For students, they analyzed eighty-eight qualitative responses to the Gillings' course evaluation question, “What will you take away from this course?” Next, the two authors familiarized themselves with the data and inductively created a thematic codebook. To ensure consistent code use, they simultaneously coded approximately twenty-five percent of transcripts, coded remaining transcripts separately, and flagged any transcripts that required further review. To identify key preceptor outcomes, the two authors analyzed the twenty-two responses to the spring end-of-semester evaluation question, “Please describe how, if at all, your organization benefited from hosting a Capstone team.” They reviewed the responses to inductively create a codebook and then worked together to apply codes to all quotations to identify thematic groups.

## Results

### Student outcomes

Since its inception in 2009, 574 students across 127 teams have completed the Capstone program, provided over 103,000 h of in-kind service, and produced more than 635 deliverables with our partner organizations. Between 2020–2022, ninety-eight students completed the current version of Capstone, provided 35,280 h of in-kind service, and produced eighty deliverables. Through our thematic analysis of course evaluation data, we identified two overarching themes for student outcomes: skill development and satisfaction.

Skill development, students' greatest takeaway from Capstone, was reflected in fifty-three percent (*n* = 47) of students' qualitative evaluation responses. Students directly named interpersonal skills (e.g., communication, teamwork, collaboration, conflict management, facilitation, community engagement, coalition building) the most. They also commented on acquisition of technical skills (e.g., project management; content development; and data collection, analysis, and reporting). In most cases, students named a mix of skills in their responses. For example, one student said they will take away:

Skills developed on the project, including survey design and implementation as well as strategies for engaging with community advisory board authentically and successfully. Shared skills among team will stick with me as well – project management, inter–team communication, strategies for setting clear expectations and holding each other accountable.

Skill development helps achieve Capstone's course aims of increasing students' capacity to address public health issues and promote health equity while enhancing their preparedness and marketability for public health careers.

Twenty-four students commented on their satisfaction with the experience when sharing key takeaways. Seven students expressed dissatisfaction, primarily with course assignments, while seventeen others remarked on their satisfaction with the experience, particularly the applied format of the course. For example, one student shared,

This Capstone project really was special. Having a community partner that demonstrated how helpful these projects would be and work with us to shape the deliverables was such a unique process. I wish we had more community–focused classes like this one.

In alignment with Capstone's objective of strengthened campus-community partnerships and CEPH ILE goals, these Capstone partnerships afford students the opportunity to see the impacts of their learning and create meaningful work that benefits external constituents.

### Community partner outcomes

Over the past 13 years, we have partnered with seventy-nine organizations representing a variety of sectors including healthcare, social services, education, and government. Twenty-five (31.6%) of our partner organizations have hosted multiple Capstone teams. Based on the twenty-two preceptor responses analyzed for this paper, two authors identified four major themes within community partner benefits: deliverable utility, enhanced capacity, broad impacts, and more inclusive processes. Sixteen (72.7%) preceptors said that they benefited from the deliverables (e.g., toolkit, communication tool, datasets, evaluation plan, report, oral history products, protocols, presentation, report, curriculum, manuscript, engagement plan) produced by their team. These findings reflect Capstone's course aim of creating new or improved public health resources, programs, services, and policies.

Fifty-seven percent (*n* = 12) of preceptors noted that project outcomes would not have been possible without the support of a Capstone team. The resources teams developed increased partner organizations' capacity to further their work. For example, a preceptor shared:

The Capstone team provided us with SO many hours of highly skilled person power that we would not otherwise have had. We now have a draft of a thorough and high quality [toolkit], which I don't think could have been created without their labor, given the resource constraints of [our organization]. This toolkit will serve as a tool to start conversations with many […] stakeholders in the future. I think it will also serve as a model for other states.

Not only can students' in-kind service and the work they produce help increase the capacity of our partner organizations, but also the Capstone project work can have long-term and far-reaching impacts for public health practice at large. Indeed, preceptors (*n* = 8) reported impacts that extend beyond the partner organization. For example, another preceptor noted,

[Our organization] will use the presentation and report that the Capstone team produced for the next decade. Not only will [our organization] benefit from advancing our strategic priorities and deepening our partnerships, but we believe this report will be used by other agencies across the county to advance behavioral health priorities in need of support.

This is an example of how Capstone can yield new and improved public health resources, programs, services, and policies that have lasting impacts beyond those directly benefiting our partner organizations.

A final theme that emerged was organizations' increased ability to implement more inclusive processes. Four preceptors commented on expanded commitment to equity initiatives as illustrated by the following quote:

The work the team did for [our organization] is work that we've talked about doing for several years - but we never had the time. The protocols are important for injured children, so we're grateful for the team's work. We also have never addressed social equity as a group. Working with this team has prompted us to take a look at our practices. The evaluation plan the students developed will provide a mechanism for us to assess and trend our implementation of the protocols and our efforts to reduce inequities in trauma care.

This example demonstrates how Capstone's commitment to working from a social change orientation can impact our partner organizations' cultures. Overall, these findings illustrate the myriad community partner benefits present within Capstone.

## Discussion

These results show that Capstone mutually benefits community partners and students. Overall, students gained skills in collaborating with communities and contributed to collective capacity to improve public health practice and tools for promoting health equity. Our finding that skill development was a key student outcome aligns with Mackenzie et al.'s (13) and Gupta et al.'s (8) evaluations of similar service-learning courses. Among skills developed, both studies cited teamwork and professional development skills as key components (8, 13). Mackenzie et al. (13), Derreth and Wear (25), and Sabo et al. (12) also report additional student outcomes that were not explicitly measured in our evaluation, including a deeper commitment to work with local communities, a deeper commitment to engaged scholarship, and stronger relationships with faculty.

In our evaluation, community partners benefitted through useful deliverables, enhanced capacity to do more public health work, impacts beyond the scope of the project, and more inclusive and equitable processes. Like our study, Gregorio et al. (23) found that their students' work products were very useful. Moreover, the Mackenzie et al. (13) study cited that students were able to offer additional capacity to organizations by “extending the[ir] reach,” which reinforced our main findings of enhanced capacity and impacts beyond the scope of the project. While not all service-learning course evaluation studies included data from community partners, our results aligned with those that did.

### Lessons learned

After 13 years, we have identified several lessons learned about implementing a critical service-learning ILE. First, despite proactive planning efforts, the teaching team has learned to expect challenges related to project scope and relationships. The solicitation and refinement of projects and partnerships starts 9 months before the beginning of Capstone. Through extended individualized support and engagement, the teaching team hopes to build trust with community partners and collaborate in shaping and strengthening their project proposals. While there are benefits of this level of engagement, no amount of planning completely insulates projects from the unforeseen challenges of community-engaged work. For example, the COVID-19 pandemic impacted how Capstone could engage with community partners, their priorities, and their staffing. In particular, preceptor turnover creates numerous challenges for team morale and project ownership, satisfaction, and impact.

Second, Capstone course assignments are designed to maximize positive experiences for students and community partners and to uphold the principles of critical service-learning, but students are often frustrated with them. The teaching team refers to the workplan and team charter as the “guardrails” of the Capstone. They exist to clarify expectations, promote power sharing and authentic relationships, and reinforce Capstone's commitment to social change. The teaching team has observed that teams who invest deeply in these documents are the least likely to encounter significant interpersonal and logistical setbacks during the experience. Despite the teaching team's messaging about the importance of these structures for mutually beneficial experiences, students routinely assert that the start of Capstone contains too much “administrative” work. While the teaching team continues to respect and incorporate students' critical feedback, they have learned to expect a certain amount of student dissatisfaction at the start of the experience.

Third, the Department has learned that having the appropriate amount of staffing and material resources to support projects is essential to ensuring positive impacts. Limiting partners to only those with material resources is one way that funding models both within public health and the non-profit sector often exclude organizations with more explicit social change agendas. Therefore, to maximize student learning and community partner benefit while minimizing community partner burden, Capstone has a high university-staff-to-project ratio and covers project expenses. To fund Capstone, the Department uses a combination of state resources and field fees. There is an enduring tension, especially because resources are scarce, to scale back spending on courses like Capstone. For experiences like these to sustain and grow, additional resources, not fewer, are needed.

Finally, programs like Capstone must adapt to shifting social, political, economic, and educational landscapes to ensure sustained positive impacts. For example, prior to the COVID-19 pandemic, the teaching team limited the eligible pool of Capstone community partners to those within a forty-mile radius of UNC-CH. The pandemic resulted in the teaching team broadening community partner eligibility criteria and now Capstone works with community partners across the nation. Capstone's expanded reach is aligned with the new vision for Public Health 3.0 where public health professionals are expected to “engage multiple sectors and community partners to generate collective impact” while improving social determinants of health (29).

### Future directions for Capstone

Public Health 3.0 (29) looks to promote health, equity, and resilience. With more community partners working on projects that explicitly tackle upstream factors like education, housing, and poverty in addition to health, Gillings will need to update its MPH training program to ensure that students enter their ILEs with the skills needed to meet these challenges. Below we describe ongoing quality improvement efforts internal to the Capstone program to strengthen outcomes for students and partner organizations.

The teaching team hopes to continue to enhance student preparedness and marketability for careers in public health. Much like other experiential learning models that report benefits to career readiness, professional leadership, and confidence (15, 18), students report a host of positive outcomes from their Capstone experience that imply preparedness and marketability. Students note the breadth and depth of technical and interpersonal skills gained, as has been reported elsewhere (13, 30). These reports of enhanced preparedness align well with findings that among undergraduate seniors seeking employment immediately after graduation, students whose course history included service-learning and capstone courses experienced greater odds of starting a new job compared with those who did not engage those high-impact practices (31). In recent years, the teaching team has offered skill-building workshops, as replicated in other programs (3), to coach students on how to present their Capstone work on résumés and how to talk about their projects during interviews using sample scripts. To simulate job applications and increase engagement with partner organizations, the teaching team will consider inviting preceptors to review and provide feedback on students' résumés and project description scripts.

The teaching team also aims to further strengthen community partnerships. One way to maximize Capstone's benefit for community partners is to adapt recruitment strategies so that the teaching team reaches more organizations for whom the Capstone experience would be most impactful. This may mean further refining the application process to lessen the time burden on potential partners and disseminating the call for Capstone projects through different channels. To enhance the experience of selected community partners, the teaching team plans to implement more preceptor-specific programming such as check-in meetings and skill-building workshops to build community and encourage collaboration among community partners.

Finally, there is a clear need for a comprehensive Capstone evaluation. The teaching team has yet to administer surveys, interviews, or focus groups that explicitly evaluate course aims and the elements of critical service learning. Furthermore, our understanding of the long-term impacts of Capstone is currently limited to anecdotal information from exchanges with former students and preceptors. By conducting a strategic evaluation, including modifications to existing course feedback opportunities and an additional alumni survey moving forward, we can better assess how Capstone is achieving course aims, operationalizing the elements of critical-service learning, and having long-term impacts.

### Recommendations for program replication

Capstone's model can be adopted or adapted by individual faculty or by schools of public health. We welcome faculty members or program and school leaders to contact us to further discuss what this might look like. In general, though, we recommend that the following core components remain consistent:

1. Program staff invest effort to ensure community partners understand the overarching goals of the experience, general timelines, logistics, and roles and responsibilities of all involved parties prior to submitting a project proposal.
2. Community partners are selected using clearly defined criteria, including equity.
3. Community partners lead the development of, and direct, students' scope of work and have flexibility in determining deliverables.
4. The experience spans two semesters (vs. something shorter like one semester or a summer).
5. Students have ample time during their assigned class time to make progress on their projects.
6. Course assignments (e.g., workplan, team charter, weekly updates) provide “guardrails” for the project experience to help ensure mutual benefit.
7. There are robust staffing supports in place to recruit and maintain community partnerships, minimize community partners' burdens, and maximize student learning. Such supports are especially important when students have nascent project management skills and limited professional experience (10, 13).

As shown in Figure 1, program staff work on Capstone activities year-round and recruit new community partners while managing a current cohort of preceptors. Clear job descriptions with timelines will be helpful in negotiations and will assist with sustainability as different faculty and staff cycle through leading this kind of experience.

### Strengths

Our description and analyses have many strengths. First, the detailed and transparent information contained in this paper will allow interested faculty to replicate and benefit from best practices found in Capstone. We openly share our course materials in the Supplementary material section and invite others to adopt or adapt these resources for their own use. Second, our results illustrate the benefits of Capstone and highlight mechanisms for ILEs to be transformative for students and community partners alike. Lastly, all authors on this paper have been members of the Capstone teaching team, students enrolled in the course, or both. This uniquely qualifies us to write this paper and share lessons learned with others in the field to advance public health training and practice.

### Limitations

As noted above, our evaluation of Capstone has some limitations. First, we designed our evaluation and analyzed data retrospectively. Therefore, evaluation tools were not explicitly aligned to our four program objectives or the elements of critical service-learning. Second, we narrowed in on qualitative data from the past 2 years instead of the past 13 years because of changes implemented in 2020. To present reflections and feedback on the current version of Capstone, we had limited data to analyze.

## Conclusion

By applying elements of critical service-learning to an ILE, Capstone is uniquely positioned to contribute to the development of public health leaders and positive community change. Community partners' project visions undergird the project selection and the course structure, which emphasizes authentic relationships, mutually beneficial processes, and practical synthesis of applied public health competencies. Through 13 years of experience, we have developed an ILE that is nimble enough to benefit community partners and rigorous enough to satisfy accreditation requirements. Capstone is a promising culminating experience practice for training skilled, collaborative public health practitioners and effecting community-driven public health change.

## Data availability statement

The data analyzed in this study is subject to the following licenses/restrictions: The data were collected for internal program evaluation. We did not request permission at the time of data collection to disseminate these raw data. Requests to access these datasets should be directed to landfried@unc.edu.

## Author contributions

ML developed the course and its content along with peer colleagues, wrote the abstract along with the learning environment, program evaluation, and results sections. ML and LS conducted the thematic data analysis. MC and LS completed a literature review, drafted the introduction and pedagogical framework section, and provided continual editing. EC wrote the discussion section and provided overall guidance for manuscript preparation. DE provided guidance, structural editing, and formatting. BP provided line edits. All authors contributed to the conception of the paper, manuscript revision, read, and approved the submitted version.
