# Supplementary material for: MPH Capstone experiences: promising practices and lessons learned
Source: Front Public Health. 2023 May 11;11:1129330. doi: 10.3389/fpubh.2023.1129330 (PMC10213715; doi:10.3389/fpubh.2023.1129330)
Supplement: Supplementary file 1 [file Table_1.DOCX]

**Supplementary Material A:** Health Equity, Social Justice, and Human Rights Required Courses and Sequencing

| Fall Semester 1 | Spring Semester 1 | Summer 1 | Fall Semester 2 | Spring Semester 2 |
| --- | --- | --- | --- | --- |
| SPHG 711: Data Analysis for Public Health (2 credits) | SPHG 721: Public Health Solutions: Systems, Policy & Advocacy (2 credits) | MPH Practicum (200 hours minimum) | SPHG 704: MPH Post-Practicum Assignments (0.5 credits) | HBEH 992: Community-Led Capstone Project II (MPH Culminating Experience) (3 credits) |
| SPHG 712: Methods and Measures for Public Health Practice (2 credits) | SPHG 722: Developing, Implementing, & Evaluating Public Health Solutions (4 credits) |  | EPID 826: Introduction to Social Epidemiology (2 credits) |  |
| SPHG 713: Systems Approaches to Understanding Public Health Issues (2 credits) | SPHG 701: Leading from the Inside-Out (2 credits) |  | PUBH 748: Leadership in Health Policy for Social Justice (3 credits) |  |
| HBEH 700: Foundations of Health Equity, Social Justice, and Human Rights (3 credits) | SPHG 703: MPH Pre-Practicum Assignments (0.5) |  | HBEH 746: Community-Led Capstone Project (3 credits) |  |
|  | HBEH 720: Leading for Racial Equity: Examining Structural Issues of Race & Class (2 credits |  |  |  |
|  | ENVR 784: Community-Driven Research and Environmental Justice (2 credits) |  |  |  |
