# Supplementary material for: MPH Capstone experiences: promising practices and lessons learned
Source: Front Public Health. 2023 May 11;11:1129330. doi: 10.3389/fpubh.2023.1129330 (PMC10213715; doi:10.3389/fpubh.2023.1129330)
Supplement: Supplementary file 2 [file Table_2.DOCX]

**Supplementary Material B:** Health Behavior Required Courses and Sequencing

| Fall Semester 1 | Spring Semester 1 | Summer 1 | Fall Semester 2 | Spring Semester 2 |
| --- | --- | --- | --- | --- |
| SPHG 711: Data Analysis for Public Health (2 credits) | SPHG 721: Public Health Solutions: Systems, Policy & Advocacy (2 credits) | MPH Practicum (200 hours minimum) | SPHG 704: MPH Post-Practicum Assignments (0.5 credits) | HBEH 992: Community-Led Capstone Project II (MPH Culminating Experience) (3 credits) |
| SPHG 712: Methods and Measures for Public Health Practice (2 credits) | SPHG 722: Developing, Implementing & Evaluating Public Health Solutions (4 credits) |  | HBEH 772: Planning, Implementing & Evaluating Health Behavior Interventions (2 credits) |  |
| SPHG 713: Systems Approaches to Understanding Public Health Issues (2 credits) | SPHG 701: Leading from the Inside-Out (2 credits) |  | HBEH 746: Community-Led Capstone Project 1 (3 credits) |  |
| HBEH 730: Theoretical Foundations of Health Behavior (3 credits) | SPHG 703: MPH Pre-Practicum Assignments (0.5 credits) |  |  |  |
| HBEH 750: Interpreting Health Behavior Research (2 credits) | HBEH 752: Health Behavior Survey Methods (2 credits) |  |  |  |
|  | HBEH 753: Qualitative Methods in Health Behavior (3 credits) |  |  |  |
