# Supplementary material for: MPH Capstone experiences: promising practices and lessons learned
Source: Front Public Health. 2023 May 11;11:1129330. doi: 10.3389/fpubh.2023.1129330 (PMC10213715; doi:10.3389/fpubh.2023.1129330)
Supplement: Supplementary file 3 [file Table_3.DOCX]

| **MPH Concentration** | **Concentration-Specific Competencies** | **CEPH MPH Foundational Competencies** |
| --- | --- | --- |
| Health Behavior | Develop, adapt, and evaluate health behavior programs and policies and scale them up. (HB Competency 5)  Engage with communities using participatory strategies and principles of effective partnerships to plan, implement, evaluate, and disseminate health behavior programs. (HB Competency 6) | Communicate audience-appropriate public health content, both in writing and through oral presentation. (Competency 19).  Integrate perspectives from other sectors and/or professions to promote and advance population health. (Competency 21). |
| Health Behavior, Social Justice, and Human Rights | Integrate relevant strategies, methodologies and measures for research, practice and policies that advance health equity, social justice, and human rights. (EQUITY Competency 2)  Incorporate cultural humility principles in public health research, practice, and policy. (EQUITY Competency 6) |  |
