# Supplementary material for: MPH Capstone experiences: promising practices and lessons learned
Source: Front Public Health. 2023 May 11;11:1129330. doi: 10.3389/fpubh.2023.1129330 (PMC10213715; doi:10.3389/fpubh.2023.1129330)
Supplement: Supplementary file 4 [file Table_4.DOCX]

**Capstone Overview**

# **Capstone Course**

Capstone (HBEH 746/992) is a year-long, community-led, group-based, mentored service-learning course that gives students in the Health Behavior (HB) and Health Equity, Social Justice, and Human Rights (EQUITY) Gillings MPH concentrations an opportunity to apply their training to public health projects in partnership with community-based organizations. As the culminating experience for the HB and EQUITY MPH concentrations, the products produced for this course serve as a substitute for The Graduate School’s master’s thesis requirement. Capstone class sessions (Tuesdays and Thursdays from 11:00 a.m.-12:15 p.m. EST) are an opportunity for students to work on, meet about, reflect on, and present their Capstone projects.

# **Capstone Project Work**

Our partner organizations define the scope of work for the Capstone projects. This approach prioritizes their specific needs and gives our students an opportunity to do applied public health work on a range of topics in a variety of settings with diverse populations. Over an entire academic year (August-April), each team of 4-6 MPH students works with a partner organization and its stakeholders to produce a set of **deliverables** (i.e., tangible products) that address one overarching goal and enhance the partner organization’s mission. A **preceptor** (i.e., organizational contact, supervisor, and mentor to the student team) outlines and supervises the project work. Each team is supported by a **faculty adviser** who provides technical expertise. The Capstone **teaching team** (i.e., course instructor(s) and teaching assistants) oversees all Capstone projects and provides structures, guidance, and supports to increase the effectiveness of the Capstone experience. As illustrated in the logic model below, we expect this service-learning experience to improve the science and tools of public health practice.


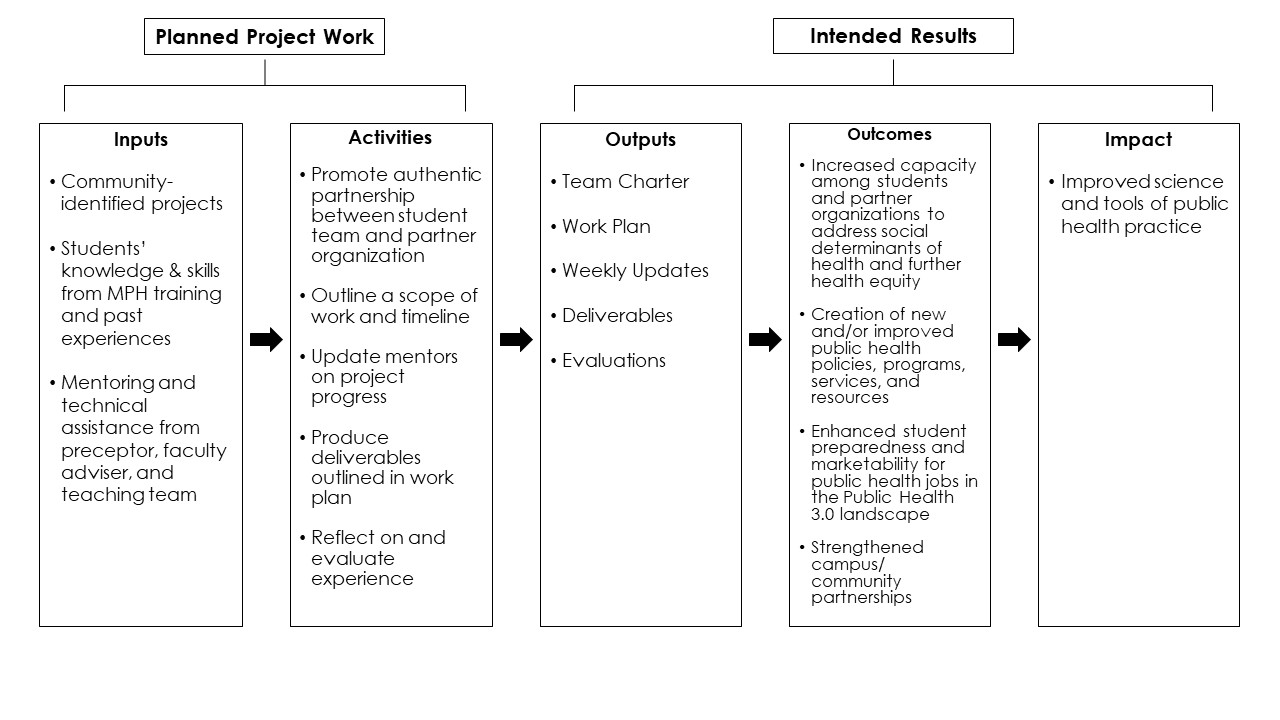


**Frequently Asked Questions**

# **What types of organizations can host a Capstone team?**

To be eligible to host a Capstone team, organizations must:

1. Be doing work that addresses a public health issue.
2. Have staff with the time (**2-4 hours per week**), expertise, and interest to mentor a team of 4-6 students.
3. Have organizational capacity and resources to sustain support for a Capstone project over the 2022-2023 academic year.

Governmental, non-governmental, non-profit, industrial, for-profit, and university-affiliated organizations are all appropriate. University-affiliated organizations must focus on community engagement, typically with external partners. **Organizations do not have to be located within North Carolina; however, preceptors must be regularly available during Capstone class time (Tuesdays and Thursdays from 11:00 a.m.-12:15 p.m. EST) to meet with their student teams.**

We prioritize organizations with demonstrated commitment to equity, inclusion, and social justice whose work allows for meaningful engagement with intended beneficiaries. Over the past 13 years, we have worked with 79 Capstone partner organizations including:

ACHIEVE Project

Alexander YMCA*

American Social Health Association

Amigas Latinas Motivando el Alma

The Art Therapy Institute*

Birth Partners UNC Medical Center Volunteer Doula Service

Boys on Track

Campus & Community Coalition to Reduce the Negative Impacts of High Risk Drinking, Chapel Hill Downtown Partnership*

Carolina Campus Community Garden

Center for Community Self-Help and Self-Help Credit Union

Cervical Cancer-Free NC

Chapel Hill-Carrboro City Schools

Chatham County Council on Aging*

Chatham County Public Health Department

Chatham Habitat for Humanity*

Children’s Home Society of North Carolina

Community Backyard

Community Transformation Grant

Counter Tobacco

Danville Regional Foundation

Duke Child and Adolescent Gender Care Clinic

Duke University Substance Abuse & Health Promotion Center

Durham County Department of Public Health

El Pueblo, Inc.*

Food Insight Group

Frank Porter Graham Bilingüe

Granville Vance Public Health

Healthy Alamance

InterFaith Food Shuttle

IntraHealth, Inc.*

Juntos

Mountain Area Health Education Center

National Implementation Research Network

NC BEAUTY Research Project

NC Cancer Hospital Comprehensive Cancer Support Program

NC Division of Public Health, Injury and Violence Prevention Branch*

NC Division of Public Health, Tobacco Prevention and Control Branch*

NC Falls Prevention Coalition

NC Latina BEAUTY

NC Poverty Research Fund

North Carolina Central University Women’s Center

North Carolina Coalition Against Domestic Violence*

North Carolina Comprehensive Cancer Program

North Carolina Division of Public Health

North Carolina Harm Reduction Coalition

Orange County Department on Aging*

Orange County Emergency Medicine Services

Orange County Health Department*

Orange County Rape Crisis Center

Orange County Solid Waste Management

Planned Parenthood of Central North Carolina

PORCH, Inc.*

PREVENT Research Project

Project IFE Research Project

Project ImPACT Research Project

Research Triangle Institute Center for Communication Science

Rural Opportunity Institute*

Sexual Health Initiatives for Teens (SHIFT) NC

Southeast Raleigh YMCA*

Southern Coalition for Social Justice

Southern Oral History Program

SpiritHouse, Inc.

State Trauma Advisory Council*

TABLE-PORCH-IFC

Toxic Free, NC

UCLA Art and Global Health Center*

UNC Center for Environmental Health and Susceptibility

UNC Community Campus Partnership

UNC Counseling and Wellness Services

UNC Department of Emergency Medicine

UNC Department of Surgery

UNC Division of General Internal Medicine

UNC Lineberger Comprehensive Cancer Center

UNC Family Medicine

UNC School of Social Work

UNC Student Wellness

UNC Trauma Program

UNC Worksite Wellness

United Way of NC

**Hosted multiple teams*

# **What are the roles and responsibilities of a preceptor?**

A preceptor is the organizational contact, supervisor, and mentor to the student team. They provide a vision for, outline, and supervise the Capstone project work. They should expect to spend **2-4 hours per week**, on average, guiding the Capstone project work. Preceptors are responsible for:

- Establishing a clear vision for the project and an appropriate and feasible scope of work that is directly aligned with the partner organization’s needs and provides a valuable learning experience for students
- Orienting students to the public health issues, people, policies, procedures, and norms related to the Capstone project work. This includes providing a guided tour of community(ies) the Capstone partner organization works with and introducing students to key informants and potential stakeholders
- Maintaining regular contact with the student team through meetings (typically every other week during Capstone class time) and email
- Providing continuous direction and specific, timely feedback on the Capstone project work based on the objectives of the project and needs of the organization
- Helping the team to problem-solve
- Modeling professional, ethical behavior
- Attending an initial team meeting (April/May 2022) orientation (August/September 2022), a spring check-in meeting (January 2023), and their team’s final presentation (April/May 2023)
- Identifying a suitable replacement if unable to continue in the role of a preceptor

# **What are the roles and responsibilities of the student team?**

Each Capstone student is expected to spend approximately **9 hours per week** working on Capstone, including both class and project work. Students are responsible for:

- Applying the knowledge and skills gained through their MPH training to the Capstone project work
- Following the steps and taking a participatory approach to producing the work outlined in the Capstone project work plan
- Managing all internal processes associated with the Capstone project work including equitable distribution of work, decision making, conflict management, etc.
- Keeping all mentors informed of support needed, decisions, and progress on the Capstone project work particularly through weekly emailed updates to preceptor(s), faculty adviser, and the teaching team
- Responding to mentor feedback
- Producing high-quality work that is useful to the Capstone partner organization and its stakeholders
- Maintaining professional and ethical behavior
- Being familiar with Capstone policies and procedures

Students should not be expected to be “extra” or replacement staff or research assistants, nor are they to provide purely administrative support.

# **What are the roles and responsibilities of the faculty adviser?**

Each team receives technical assistance from a faculty adviser who is responsible for:

- Maintaining regular contact and communication with the Capstone team by at a minimum responding as needed to weekly updates and meeting with team ~3 times per semester
- Providing specific, timely feedback to students about their quality of work and performance
- Ensuring that students’ work meets the level of quality expected for a master’s thesis substitute
- Grading Capstone deliverables and assessing students’ demonstration of competency attainment
- Attending an initial team meeting (April/May 2022) orientation (August/September 2022), a spring check-in meeting (January 2023), and their team’s final presentation (April/May 2023)

# **What are the roles and responsibilities of the teaching team?**

The teaching team oversees the Capstone program. They are responsible for:

- Communicating expectations for the Capstone program
- Providing guidance on Capstone processes and project work
- Managing stakeholder (e.g., students, preceptors, faculty advisers, accreditors) interests and priorities
- Facilitating feedback and problem solving among Capstone parties
- Promoting consistency across the Capstone projects’ depth, scope, and quality

# **What conditions promote Capstone project success?**

Capstone projects are most successful for **organizations** when:

- The preceptor has a clear vision for the project work
- There is an immediate need and use for the deliverables produced
- The work is informed by and is responsive to project stakeholders, including those most impacted by the project topic
- Leadership at the Capstone partner organization fully supports the Capstone project and provides the resources (including staff time) necessary to carry out the project work

Capstone projects are most successful for **students** when:

- The preceptor provides clear direction for the project work
- The project work allows students to apply and develop a wide range of skills that will enhance their growth as public health practitioners
- Deliverables have clear purposes, are interrelated, and serve one overarching project goal
- The preceptor has dedicated time, expertise, and interest to mentor graduate students
- There is ample time and attention given to orienting students to the project work, partner organization, and key stakeholders, including the project’s intended beneficiaries
- They have an opportunity to interact with the populations most impacted by the project work

# **What types of deliverables do Capstone teams produce?**

Each Capstone team typically produces 4-6 major deliverables. Examples include:

- Assessment Tools
- Community Assessment Reports
- Curricula
- Dissemination Plans
- Evaluation Plans
- Evaluation Tools
- Evidence Tables
- Focus Group Guides
- Formative Research Findings Reports
- Funding Guides
- Grant Proposals
- Health Communication Campaign Materials
- Health Communication Plans
- Intervention Materials
- Interview Guides
- IRB Applications
- Literature Reviews
- Manuscripts
- Message Testing Reports
- Photovoice Projects
- Policies and Procedures Manuals
- Policy Briefs
- Policy Recommendations
- Presentation Materials
- Program Plans
- Sustainability Plans
- Training Workshop Materials
- Video Storyboards

# **Can a Capstone team work remotely?**

Yes. Capstone teams do not have to work onsite at an organization. However, Capstone students must have opportunities to ethically engage with the people most directly impacted by their work. We have a limited budget to support local travel as a part of Capstone activities.

# **Is there a fee associated with having a Capstone team?**

No. There is no charge for the services Gillings students, faculty, and staff provide during this mentored field experience. Annually, our Capstone students provide approximately $255,000 of in-kind service^[[1]](#footnote-2)^ to our partner organizations.

# **How do I apply for a Capstone team?**

To apply for a Capstone team, please follow these steps:

1. Review the Capstone Overview document in full.
2. Contact [mphcapstone@unc.edu](mailto:mphcapstone@unc.edu) to schedule a meeting with a member of the Capstone teaching team to discuss your project ideas and receive tips on the Capstone project proposal process. **Meetings with the teaching team should take place by January 31, 2022**.
3. Fill out the 2022-2023 Capstone Project Proposal Form, available [here](https://sph.unc.edu/hb/hb-capstone/). Please save your completed proposal as a **Microsoft Word** document using the following naming convention: Organization Name_Capstone Proposal 2022-2023.
4. Collect a letter of support from your organization’s leadership. The letter of support should explain how the Capstone project goal will enhance your organization’s mission; demonstrate commitment to the resources and support (including staff time) needed to carry out the project work; and outline a contingency plan if the preceptor is no longer able to carry out the roles and responsibilities of that position.
5. Email your completed proposal form and a letter of support to [mphcapstone@unc.edu](mailto:mphcapstone@unc.edu) by 11:59 p.m. on **February 7, 2022**.

# **If my organization applies for a Capstone team, are we certain to get one?**

No. Given the varied interests and expertise of our students, there is no guarantee that a proposal will be selected. If your project is not selected, either at the proposal review stage or through student rankings, you may be encouraged to apply in a future year or to reconsider the scope of your project proposal to take advantage of other field training opportunities such as the [practicum](https://sph.unc.edu/resource-pages/master-of-public-health/mph-practicum-2/)).

# **How are Capstone projects selected?**

Submitted proposals are reviewed by a committee of faculty and current students. Proposals are scored based on the following criteria:

- **Project Clarity**: The proposed deliverables have clear purposes and steps, are interrelated, and connect to the overall project goal.
- **Project Feasibility**: The breadth and depth of deliverables and proposed timeline is appropriate for 4-6 students to produce over two academic semesters (August-April while classes are in session). The proposal accounts for the time and effort needed to onboard students.
- **Learning Opportunities**: The project will facilitate acquisition of knowledge and skills that will enhance students’ growth as public health practitioners.
- **Mentorship**: The preceptor has the time (2-4 hours per week), expertise, and interest needed to mentor MPH students.
- **Organizational Capacity**: The partner organization has capacity and funding to sustain support for a Capstone project over the 2022-2023 academic year. Leadership at the Capstone partner organization demonstrates full support of the Capstone project. Sustainability and contingency plans are clear and feasible.
- **Approach**: The Capstone partner organization demonstrates commitment to equity, inclusion, and social justice in their approach to addressing public health problems. The project work is designed to be equitable and sustainable.
- **Engagement**: The project work is informed by and responsive to project stakeholders including those most directly impacted by the issue. Students will have an opportunity to interact with the intended beneficiaries of the project work.
- **Public Health Impact**: The project has strong potential to make a meaningful difference in the health of the beneficiary communities and population(s).

We will invite preceptors whose proposals receive the highest scores to submit a brief (~5 minute) recorded project overview presentation that will be shared with students. After reviewing the submitted presentations, students will rank their top five choices for Capstone projects. The Capstone teaching team will then conduct a matching process among the projects, students, and faculty advisers. Prospective organizations will be notified whether their projects have been selected to receive a Capstone team in early April.

# **What is the timeline for the 2022-2023 Capstone projects?**

The Capstone solicitation, selection, and matching process will occur between December 2021 and April 2022. Capstone teams (students, preceptor, faculty adviser) are expected to meet with the teaching team once before the 2022 summer break to review roles and responsibilities and outline a plan for onboarding the student team to the project work and partner organization. Capstone project work will officially begin in August 2022 and will continue while classes are in session until April 2023. The Capstone project work will culminate with final presentations in late April 2023.

| **Date** | **Task** | **Who’s Responsible** |
| --- | --- | --- |
| December 2021-  January 2022 | Solicit potential Capstone projects | Capstone Teaching Team |
| January-early February 2022 | Collect Capstone project proposals | Capstone Teaching Team |
| January-February 2022 | Review and rank Capstone project proposals | Capstone Project Selection Committee |
| February 2022 | Select projects to be “pitched” to students in March | Capstone Project Selection Committee |
| TBD March 2022 | Present project proposal at Capstone Pitch Day | Selected Preceptors |
| March 2022 | Rank top 5 choices for Capstone projects | Students |
| March 2022 | Facilitate matching process between students, Capstone projects, and faculty advisers | Capstone Teaching Team |
| By April 3, 2022 | Announce 2022-2023 Capstone projects, preceptors, student teams, and faculty advisers | Capstone Teaching Team |
| April/May 2022 | Conduct initial Capstone team meetings | Students, Preceptors, Faculty Advisers, Capstone Teaching Team |
| June 1, 2022 | Submit signed draft work plan | Students |
| Late August 2022 | Attend Capstone Orientation | Students, Preceptors, Faculty Advisers |
| Late August/early September 2022 | Orient students to Capstone partner organization | Preceptors |
| September 2022 | Finalize fall work plan | Students, Preceptors, Faculty Advisers |
| January 2023 | Attend spring check-in meetings | Students, Preceptors, Faculty Advisers, Capstone Teaching Team |
| January 2023 | Update work plan | Students, Preceptors, Faculty Advisers |
| Late April 2023 | Turn in completed deliverables | Students |
| Early May 2023 | Present results and impact of Capstone work | Students, Preceptors, Faculty Advisers |

# **Whom should I contact if I have questions?**

Please contact [mphcapstone@unc.edu](mailto:mphcapstone@unc.edu).

1. Calculation based on: average of 45 students/year x 9 hours of Capstone work/week/student x 30 weeks/year x $20.96 (graduate assistant hourly rate) = $254,664. [↑](#footnote-ref-2)
