# Supplementary material for: MPH Capstone experiences: promising practices and lessons learned
Source: Front Public Health. 2023 May 11;11:1129330. doi: 10.3389/fpubh.2023.1129330 (PMC10213715; doi:10.3389/fpubh.2023.1129330)
Supplement: Supplementary file 5 [file Table_5.DOCX]

#
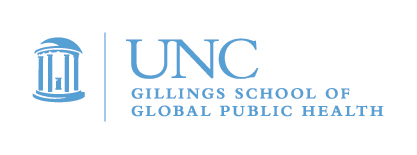


**Capstone Project Proposal Form**

**Instructions**

1. Review the Capstone Overview document in full.
2. Contact [mphcapstone@unc.edu](mailto:mphcapstone@unc.edu) to schedule a meeting with a member of the Capstone teaching team to discuss your project ideas and receive tips on the Capstone project proposal process. Meetings with the teaching team should take place by **January 30, 2023**.
3. Fill out this form. Please save your completed proposal as a **Microsoft Word** document using the following naming convention: Organization Name_Capstone Proposal 2023-2024.
4. Collect a letter of support from your organization’s leadership. The letter of support should explain how the Capstone project goal will enhance your organization’s mission; demonstrate commitment to the resources and support (including staff time) needed to carry out the project work; and outline a contingency plan if the preceptor is no longer able to carry out the roles and responsibilities of that position.
5. Email your completed proposal form and a letter of support to [mphcapstone@unc.edu](mailto:mphcapstone@unc.edu) by 11:59 p.m. on **February 6, 2023**.

**Partner Organization**

Organization:

Address:

Website:

*Please provide a description of your organization.*

*How is your organization committed to equity, inclusion, and social justice in its approach to addressing public health problems?*

*Please explain why your organization would benefit from working with a team of MPH students.*

**Preceptor(s)**

*Who will be the preceptor (i.e., organizational contact, supervisor, and mentor) to the student team?*

Name, Degree(s):

Job Title:

Email:

Phone:

The most successful Capstone experiences have a preceptor who remains involved from the application process through project completion. Preceptor continuity helps ensure projects stay on track and promotes authentic relationships between partner organizations and student teams. To the best of your knowledge, do you plan to stay in your role with the ability to serve as the primary preceptor for the entire academic year? **Please confirm your commitment to serving as a preceptor by checking the box next to the statement below.**

I acknowledge that Capstone is a year-long commitment and, to the best of my knowledge, plan to stay in my role and serve as the primary preceptor for the entire year.

*If your proposed project would best be supported by more than one preceptor, please provide contact information for a second preceptor and a rationale for having more than one preceptor.*

Name, Degree(s):

Job Title:

Email:

Phone:

Rationale for having more than one preceptor:

**Capstone Project Title**

*Provide a 1-sentence title that summarizes the primary activities and goal of the project (e.g., Evaluation of a Dual-Method Campaign to Prevent STIs and Pregnancy and Promote PrEP Use Among North Carolina Youth).*

**Capstone Project Overview**

*Provide a <1 page description of your project that includes:*

- *An explanation of the public health issue(s) prompting the need for the Capstone project*
- *An overview of the major activities a team will conduct, the deliverables they will produce, and the intended impact of their work*
- *How the proposed Capstone project work will build on, complement, or differ from past Capstone projects with your organization (if applicable)*

**Capstone Project Deliverables**

*We expect each Capstone team to produce 4-6 major deliverables (i.e., products) for their Capstone partner organization. For each deliverable:*

- *Provide a deliverable title that clarifies the type of product students will produce (e.g., literature review, interview guide, evaluation plan, curriculum, funding guide, etc.).* ***Deliverable titles must be nouns.***
- *Describe the purpose of the deliverable by explaining* ***why*** *the product is needed. A deliverable’s purpose should not be a summary of the activities the students will complete when producing the deliverable but rather a clarification of the deliverable’s intended impact.*
- *Describe the expected format of the deliverable. Here it is helpful to note the expected length and formality of the deliverable (e.g., a 20 page academic literature review vs. a ~5-page annotated bibliography).*
- *List the audience(s) for the deliverable (e.g., organizational leadership, community members, policy makers, funders, etc.).*
- *Provide a list of the major steps needed to produce the deliverable as well as an estimated timeline.*

***Example:***

| **Deliverable #1: Survey Tool** | |
| --- | --- |
| *Purpose:* To collect information from service providers regarding the availability of services and barriers to access, particularly regarding the Capstone partner’s priority populations. | |
| *Format:* Qualtrics tool and a Word document with survey items | |
| *Intended Audience:* preceptor, community stakeholders | |
| **Steps** | **Expected Completion Date** |
| 1. Meet with preceptor re: ideas for the organizational capacity survey | 9/8/2023 |
| 1. Begin drafting items for the organizational assessment – this includes the different sections of the survey, general ideas for questions, and survey objectives in preparation for the CAB meeting. | 9/8/2023 |
| 1. Present ideas for research and survey questions to preceptor and get initial feedback | 9/15/2023 |
| 1. Complete first draft of research and survey questions | 9/21/2023 |
| 1. Receive feedback and make edits on first draft to prepare for presentation to CAB | 9/29/2023 |
| 1. Present organizational survey draft at CAB meeting | 9/30/2023 |
| 1. Collect TA feedback on organizational survey draft. | 10/6/2023 |
| 1. Co-organize and co-lead Organizational Survey Retreats with Research Team and CAB to get feedback on survey draft. | 10/20/23 |
| 1. Collaborate with the preceptor on a draft IRB (preceptor will be responsible for the IRB, but this is an opportunity for students to engage in the process) | 11/05/2023 |
| 1. Use an iterative feedback process with the research team and the CAB to confirm the data collection tool | 11/09/23 |
| 1. Present survey recruitment plan, sampling frame, and current draft of organizational survey to CAB meeting for additional feedback. | 11/11/23 |
| 1. Incorporate final CAB feedback and make decisions on feedback received. Incorporate edits into organizational survey to finalize draft for IRB approval. | 12/1/23 |
| 1. Finalize survey recruitment and sampling strategy. | 12/1/2023 |
| 1. Finalize the survey tool in Qualtrics. This deadline will reflect a tentative Jan. 1^st^ start time for data collection. | 12/31/2023 |
| 1. Submit deliverable to preceptor, faculty adviser, TA, and instructor per deliverables assignment instructions. | 4/21/2024 |

*Please remove the above example from your finalized Capstone project proposal form.*

| **Deliverable #1: Deliverable Title (Must be a Noun)** | |
| --- | --- |
| *Purpose:* | |
| *Format:* | |
| *Intended Audience:* | |
| **Steps** | **Expected Completion Date** |
|  |  |
|  |  |
|  |  |
|  |  |
|  |  |
|  |  |

| **Deliverable #2: Deliverable Title (Must be a Noun)** | |
| --- | --- |
| *Purpose:* | |
| *Format:* | |
| *Intended Audience:* | |
| **Steps** | **Expected Completion Date** |
|  |  |
|  |  |
|  |  |
|  |  |
|  |  |
|  |  |

| **Deliverable #3: Deliverable Title (Must be a Noun)** | |
| --- | --- |
| *Purpose:* | |
| *Format:* | |
| *Intended Audience:* | |
| **Steps** | **Expected Completion Date** |
|  |  |
|  |  |
|  |  |
|  |  |
|  |  |
|  |  |

| **Deliverable #4: Deliverable Title (Must be a Noun)** | |
| --- | --- |
| *Purpose:* | |
| *Format:* | |
| *Intended Audience:* | |
| **Steps** | **Expected Completion Date** |
|  |  |
|  |  |
|  |  |
|  |  |
|  |  |

**Engagement, Skills, & Success**

*Please describe how the student team will engage with the intended beneficiaries of the Capstone project work.*

*Please list the knowledge/skills/expertise that students should have prior to beginning the Capstone project work (e.g., familiarity with xyz topic, qualitative data collection, experience with a particular method or software, etc.).*

| Required Knowledge/Skills/Expertise | Nice-to-Have Knowledge/Skills/Expertise |
| --- | --- |
|  |  |

*Please list the technical, professional, and/or interpersonal skills you expect students to gain by working on this Capstone project.*

*What are the major challenges for getting this Capstone project accomplished? How will the preceptor and Capstone partner organization support the student team in navigating those challenges?*

*Please describe what success for this project looks like.*

**Mentorship**

*Please confirm that the preceptor(s) listed on page 1 agrees to (check boxes):*

Spend approximately 2-4 hours, on average, each week supporting the Capstone project work.

Be regularly available on **Tuesdays and/or Thursdays from 11:00-12:15 EST** to meet with their Capstone student team.

Establish a clear vision for the project and an appropriate and feasible scope of work that is directly aligned with the partner organization’s needs and provides a valuable learning experience for students.

Orient students to the public health issues, people, policies, procedures, and norms related to the Capstone project work. This includes providing a guided tour of community(ies) the Capstone partner organization serves/intends to serve (if applicable) and introducing students to key informants and potential stakeholders.

Maintain regular contact with the student team through meetings (typically every other week) and email.

Provide continuous direction and specific, timely feedback on the Capstone project work based on the objectives of the project and needs of the organization.

Help the team to problem-solve.

Model professional, ethical behavior.

Attend an initial team meeting (April/May 2023) orientation (August/September 2023), a spring check-in meeting (January 2024), and their team’s final presentation (April/May 2024).

Identify a suitable replacement if unable to continue in the role of a preceptor.

*Please describe the preceptor’s experience with and approach to mentoring graduate students.*

*Please describe the expected format (e.g., in person, online) and frequency of interactions between the student team and preceptor.*

**Special Considerations**

*What are important timing considerations (e.g., deadlines, events) for the student team to be aware of when working on the proposed project? See* [*UNC Academic Calendar*](https://registrar.unc.edu/wp-content/uploads/sites/9/2022/09/Chancellor-Calendar-AY21-22-22-23.pdf) *for students’ availability.*

*What, if any, travel considerations exist for students who will work on this project? Please describe the purpose of the travel as well as any details about anticipated timing and duration (e.g., To collect key informant data, the Capstone team will be expected to travel to xyz location three times in the fall semester. Each trip will take approximately # hours and will occur on a weekday).*

*What, if any, language considerations exist for students who will work on this project? Please explain whether students will be required to conduct work in another language and/or work with a translator and/or interpreter.*

*What financial resources/costs do you expect this project to require? What, if any, resources from your organization will be used to cover those expenses?*

*Federal regulations require that research projects involving human subjects be reviewed by an* [*Institutional Review Board*](https://research.unc.edu/human-research-ethics/about/) *(IRB). To help us understand whether your proposed project will need an IRB, please indicate whether any of the following statements are true:*

The proposed project meets the definition of research (i.e., *systematic* investigation, including research development, testing, and evaluation, designed to develop or contribute to *generalizable* knowledge)

The proposed project will include data collection from human subjects (i.e., a living individual about whom an investigator [professional or student] conducting research: (i) Obtains information or biospecimens through intervention or interaction with the individual, and uses, studies, or analyzes the information or biospecimens; or (ii) Obtains, uses, studies, analyzes, or generates identifiable private information or identifiable biospecimens).

*How will the project work be sustained after the academic year ends?*

*What other important issues, if any, should the Capstone students, faculty adviser, and/or Capstone teaching team know about your proposed project?*

**Please return completed proposal forms to** [**mphcapstone@unc.edu**](mailto:mphcapstone@unc.edu)**.**
