# Supplementary material for: MPH Capstone experiences: promising practices and lessons learned
Source: Front Public Health. 2023 May 11;11:1129330. doi: 10.3389/fpubh.2023.1129330 (PMC10213715; doi:10.3389/fpubh.2023.1129330)
Supplement: Supplementary file 6 [file Table_6.docx]

# Capstone Partner Organization Name

# Team Charter

# 2022-2023

## Capstone Team Students

| Picture  **First Name Last Name**  (pronunciation tips)  Concentration  Capstone Role (e.g., teaching team liaison, preceptor liaison, faculty adviser liaison, etc.)  *Phone:* (XXX) XXX-XXXX  *Email: UNC email address* | To help the teaching team, your preceptor(s), and your faculty adviser get to know you better, add a brief biosketch here. |
| --- | --- |
| Picture  **First Name Last Name**  (pronunciation tips)  Concentration  Capstone Role (e.g., teaching team liaison, preceptor liaison, faculty adviser liaison, etc.)  *Phone:* (XXX) XXX-XXXX  *Email: UNC email address* | To help the teaching team, your preceptor(s), and your faculty adviser get to know you better, add a brief biosketch here. |
| Picture  **First Name Last Name**  (pronunciation tips)  Concentration  Capstone Role (e.g., teaching team liaison, preceptor liaison, faculty adviser liaison, etc.)  *Phone:* (XXX) XXX-XXXX  *Email: UNC email address* | To help the teaching team, your preceptor(s), and your faculty adviser get to know you better, add a brief biosketch here. |
| Picture  **First Name Last Name**  (pronunciation tips)  Concentration  Capstone Role (e.g., teaching team liaison, preceptor liaison, faculty adviser liaison, etc.)  *Phone:* (XXX) XXX-XXXX  *Email: UNC email address* | To help the teaching team, your preceptor(s), and your faculty adviser get to know you better, add a brief biosketch here. |
| Picture  **First Name Last Name**  (pronunciation tips)  Concentration  Capstone Role (e.g., teaching team liaison, preceptor liaison, faculty adviser liaison, etc.)  *Phone:* (XXX) XXX-XXXX  *Email: UNC email address* | To help the teaching team, your preceptor(s), and your faculty adviser get to know you better, add a brief biosketch here. |

## Capstone Team Mentors

| **First Name Last Name**  (pronunciation tips)  Preceptor | *Phone:* (XXX) XXX-XXXX  *Email:* |
| --- | --- |
| **First Name Last Name**  (pronunciation tips)  Faculty Adviser | *Phone:* (919) XXX-XXXX  *Email: UNC email address* |
| **First Name Last Name**  (pronunciation tips)  Teaching Assistant | *Phone:* (919) XXX-XXXX  *Email: UNC email address* |

## Roles & Responsibilities

### Students

Students produce the Capstone work. Each Capstone student is expected to spend **6-9 hours per week outside of class time** working on Capstone, including both project work and course assignments. Students are responsible for:

- Applying the knowledge and skills gained through their MPH training to the Capstone project work
- Following the steps and taking a participatory approach to producing the work outlined in the Capstone project work plan
- Managing all internal processes associated with the Capstone project work including equitable distribution of work, decision making, conflict management, etc.
- Keeping their TA, preceptor(s), faculty adviser, and their instructorinformed of support needed, decisions, and progress on the Capstone project work
- Seeking and responding to TA, preceptor, and faculty adviser feedback
- Producing high-quality work that is useful to the Capstone partner organization and its stakeholders
- Maintaining professional and ethical behavior
- Being familiar with Capstone policies and procedures
- Identifying a *preceptor liaison* who is responsible for:
  - Being the main point of contact for the preceptor
  - Making requests for preceptor support (outside of Weekly Updates)
  - Fielding needs, questions, and feedback from the preceptor
- Identifying a *faculty adviser liaison* who is responsible for:
  - Being the main point of contact for the faculty adviser
    - - - Making requests for faculty adviser support (outside of Weekly Updates)
        - Fielding needs, questions, and feedback from the faculty adviser
- Identifying a *teaching team liaison* who is responsible for:
  - - - - Sending the team’s Weekly Update per assignment instructions
        - Turning in group course assignments
  - Being the main point of contact for the teaching team
    - - - Making requests for teaching team support
        - Fielding needs, questions, and feedback from the teaching team
- Identifying a *deliverable lead* for each deliverable who is responsible for:
  - Coordinating deliverable logistics (e.g., securing resources needed)
  - Ensuring that the team follows the steps and timeline listed in the deliverable table
  - Ensuring consistent quality, voice, and formatting within the deliverable

Students should not be expected to be “extra” or replacement staff or research assistants, nor are they to provide purely administrative support.

### Preceptors

Preceptors create a vision for, direct, and supervise the Capstone project work. They should expect to spend **2-4 hours per week**, on average, guiding the Capstone project work. Preceptors are responsible for:

- Establishing a clear vision for the project and an appropriate and feasible scope of work that is directly aligned with the partner organization’s needs and provides a valuable learning experience for students
- Orienting students to the public health issues, people, policies, procedures, and norms related to the Capstone project work. This includes providing a guided tour of community(ies) the Capstone partner organization works with and introducing students to key informants and potential stakeholders
- Maintaining regular contact with the student team through meetings (typically every other week) and email (especially weekly updates)
- Providing continuous direction and specific, timely feedback on the Capstone project work based on the objectives of the project and needs of the organization
- Helping the team to problem-solve
- Modeling professional, ethical behavior
- Attending an initial team meeting (April/May 2022), orientation (August/September 2022), a spring check-in meeting (January 2023), and their team’s final presentation (April/May 2023)
- Identifying a suitable replacement if unable to continue in the role of a preceptor

### Faculty Adviser

Faculty advisers provide technical assistance and quality assurance for the Capstone project work. They should expect to spend **1-2 hours per week**, on average, providing feedback and guidance. Each team receives support from a faculty adviser who is responsible for:

- Maintaining regular contact and communication with the Capstone team by at a minimum responding to weekly updates and meeting with team ~3 times per semester
- Providing technical expertise
- Providing specific, timely feedback to students about their quality of work and professionalism
- Ensuring that students’ work meets the level of quality expected for a master’s thesis substitute
- Grading Capstone deliverables and assessing students’ demonstration of competency attainment
- Attending an initial team meeting (April/May 2022), orientation (August/September 2022), a spring check-in meeting (January 2023), and their team’s final presentation (April/May 2023)

## Team Values

| Value | Behavior |
| --- | --- |
| To establish an intentional culture within your team, list the values (one per row, max 5) your team wants to uphold to promote authentic partnership between Capstone students, the preceptor(s), and the faculty adviser. | To clarify how you will operationalize the value, describe the behavior(s) you will engage in to uphold each value. |
|  |  |
|  |  |
|  |  |

## Accountability Plan

To take responsibility for upholding the team’s desired culture, describe how you will hold one another accountable to staying aligned with the agreed upon values.

## Team Processes

### Meetings

To clarify expectations for team meetings, at a minimum, document:

- Your team’s views on characteristics of an effective meeting
- Meeting frequency/format for:
  - Just the student team (teams should expect to meet outside of Capstone class time)
  - Students + preceptor (most teams meet with their preceptor at least every other week)
  - Students + faculty adviser (most teams meet with their faculty adviser once a month)
- How you will share power and promote equitable contributions to, and participation in, team meetings

### Project Management

To clarify the tools and strategies you will use for producing your deliverables, at a minimum, document:

- What tools/technologies you will use to work together most effectively
- How you will capitalize on strengths and different work styles among team members
- How you will promote equitable contributions to the Capstone project work
- How you will ensure consistent quality and voice within and across deliverables
- How will you know if your progress on steps between milestones is adequate to reach the larger goals

### Communication

To clarify communication expectations, at a minimum, come to consensus and document:

- Preferred communication formats (Zoom, Email, MS Teams, GroupMe, etc.)
- Expected response times to Capstone communications and procedures for following up if a response is not received for communications:
  - Within the student team
  - With your preceptor
  - With your faculty adviser
- Expectations for giving and receiving feedback
- Who will serve in the following liaison roles (see student roles and responsibilities for more information):
  - Preceptor Liaison:
  - Faculty Adviser Liaison:
  - Teaching Team Liaison:

### Decision Making

To clarify decision making processes, at a minimum, document:

- What decision-making approaches your team will use
- What strategies you will employ for navigating decisions that need to be revisited or ones that are not honored

### Conflict Management

To clarify how you will handle conflict when it arises, at a minimum, document:

- What strategies your team will employ to proactively address conflict
- How you will name hurt when it happens
- What repairing mistakes looks like for the team
- What steps the team will take if conflict is not resolved after addressing it

### Support & Celebration

To cultivate positive relationships between Capstone students, your preceptor(s), and your faculty adviser, at a minimum, document how you will:

- Build community within your team
- Encourage and support personal and professional growth
- Celebrate accomplishments

## Agreement

I agree to my roles and responsibilities as team member and commit to the guiding principles and processes of our partnership. This Team Charter may be amended with the consent of all parties named below.

| Name | Role |
| --- | --- |
| [insert name here] | Student |
| [insert name here] | Student |
| [insert name here] | Student |
| [insert name here] | Student |
| [insert name here] | Preceptor |
| [insert name here] | Faculty Adviser |
| [insert name here] | Teaching Assistant (TA) |
| [insert name here] | Instructor |
