# Supplementary material for: MPH Capstone experiences: promising practices and lessons learned
Source: Front Public Health. 2023 May 11;11:1129330. doi: 10.3389/fpubh.2023.1129330 (PMC10213715; doi:10.3389/fpubh.2023.1129330)
Supplement: Supplementary file 7 [file Table_7.docx]

# Capstone Partner Organization Name

# Workplan

# 2022-2023

## Project Title

As the substitute for the Graduate School’s master’s thesis requirement, your Capstone project must have a title for graduation paperwork. List one here. Your title should summarize the primary activities of your work. For example:

- *Implementation, Evaluation, and Resource Development for Chatham County Council on Aging’s Community Ambassador Program*
- *Evaluation of a Dual-Method Campaign to Prevent STIs and Pregnancy and Promote PrEP Use Among North Carolina Youth*
- *Sexual Health Education for LatinX Youth: A Cultural Adaptation of the Wise Guys Curriculum*
- *Adapting an HIV Prevention Intervention for African American Women Living in Public Housing Communities in Durham, NC*
- *Identifying Smoke-Free Air Policy and Implementation Best Practices to Strengthen the Durham County Board of Health Smoking Rule*

Simply listing your Capstone partner organization’s name is insufficient for graduation paperwork.

## Deliverables

Using the deliverable tables included in your Capstone partner organization’s project proposal as a guide, fill out the template below for each deliverable you are expected to produce:

| **Deliverable 1:** To clarify the tangible output of your work, list a deliverable title here. Deliverables are what serve as the technical substitute for the master’s thesis requirement and as such their titles must be nouns that describe the tangible outputs of the steps listed. E.g., Literature Review, Data Collection Instrument, Recommendations Report, etc. | |
| --- | --- |
| *Purpose:* To ensure all parties understand the intended impact of the deliverable, explain why the deliverable is being produced here. | |
| *Intended Audience:* To ensure the deliverable is designed for the appropriate audience, list the people/organization(s) who will use the deliverable here. | |
| *Format:* To ensure all parties know what type of product will best serve the intended audience, describe what the deliverable should look like (i.e., document type, length, level of formality, etc.) here. | |
| *Deliverable Lead:* To create efficiencies for managing the deliverable, list the name of the student who will:   - Coordinate deliverable logistics (e.g., secure resources needed) - Manage deliverable progress - Ensure consistent quality, voice, and formatting within the deliverable | |
| **Steps** | **Expected Completion Date** |
| 1. To ensure that you have a clear plan of action that is appropriate and feasible for the timeline, list all steps (one step per row) you will take to produce the deliverable. If applicable to the deliverable, steps listed should account and budget a reasonable amount of time for:  - Getting oriented to the partner organization/subject matter (e.g., reading background materials, conducting a windshield tour of the community, meeting key stakeholders) - Applying for IRB approval - Outlining materials (to ensure all parties agree to the direction of the deliverable) - Drafting materials - Soliciting multiple rounds of feedback from Capstone mentors (i.e., TA, preceptor, faculty adviser) and key stakeholders. **NOTE: We expect your TA, preceptor(s), and faculty adviser(s) to provide feedback on your work throughout the process of developing your deliverables.** We encourage you to be thoughtful about how to best utilize your mentors based on the following roles:   - TA: Helps strengthen the polish of the work and directs students to resources. We strongly encourage you to involve TAs in brainstorming in shaping the work and submit work to your TA for review before submitting to your preceptor and faculty adviser to reduce their workloads.   - Preceptor: Provides guidance on what content and format is most appropriate for their needs.   - Faculty adviser: Provides technical assistance and quality control. - Piloting - Recruiting participants/respondents - Analyzing data - Finalizing materials - Disseminating materials (i.e., how the deliverable will be presented/handed off and to whom) - Building capacity/planning for sustainability (i.e., ensuring the partner organization will be able to use the deliverable once the Capstone team finishes their work)   NOTE: For consistency and ease of review, steps should begin with **verbs** (e.g., outline, draft, collect, finalize, etc.) | For steps due in the fall, list a specific date (e.g., October 3). Until December 2, you may list a month for steps due in the spring. By December 2, all deliverable steps should have specific due dates. |
|  |  |
|  |  |
|  |  |
|  |  |
|  |  |
|  |  |
| 1. Submit deliverable to preceptor, faculty adviser, TA, and instructor per deliverables assignment instructions. | April 20, 2023 |

## Key Dates

To help everyone keep course deadlines in mind, we’ve listed key dates from the HBEH 746 syllabus below. We will share Spring 2023 key dates later in the semester. The teaching team reserves the right to make changes to the syllabus, including how we use class time and assignment due dates. Changes to the syllabus/assignments will be communicated as early as possible and it is the student team’s responsibility to ensure that the dates listed below match the syllabus. **Please add any other project-specific key dates (e.g., key constituent meetings, preceptor travel, organizational deadlines) that could impact your deliverables to the table below:**

| Assignment/Task/Event | Date |
| --- | --- |
| Team Meeting to Agree on Team Charter & Work Plan | August 30 |
| Draft Team Charter & Work Plan Due | September 1 |
| Check-In Meeting with Instructor and TA (Fall 1) | Add date/time from syllabus |
| Draft Project Summary Visual & Script Due | September 22 |
| Reflection Session I | September 27 |
| Fall Mid-Semester Evaluation Due | October 6 |
| Check-In Meeting with Instructor and TA (Fall 2) | Add date/time from syllabus |
| Check-In Meeting with Instructor and TA (Fall 3) | Add date/time from syllabus |
| Updated Team Charter Due | November 17 |
| Updated Project Summary Visual & Script Due | November 17 |
| Reflection Session II | November 29 |
| Updated Work Plan and Fall End-of-Semester Evaluation Due | December 1 |

## Deliverable Ownership

Capstone Partner Organization Name owns the final deliverables. However, the Department of Health Behavior reserves the right to publicly list Capstone Partner Organization Name as a Capstone partner, to keep copies of all Capstone teams' final deliverables for review by the Gillings School of Global Public Health community, and to include a brief project description in Capstone promotional materials.

Each Capstone student will maintain a copy of the final deliverables to use as evidence of their work on the project for potential job interviews and as potential writing samples or portfolio pieces. The Department of Health Behavior and the Capstone students will seek permission if they wish to use the deliverables for any purpose other than the one stated above.

## Authorship

Tailor the following language to your circumstances:

Any public documents that result from this project will include names of Capstone team members who provided meaningful contributions to the work as authors. The student team and Capstone Partner Organization Name will come to an agreement on what constitutes authorship for each deliverable as well as the order in which authors will be listed.

**Use of Recorded Materials**

All recorded materials will be used and disposed of according to Institutional Review Board approved methods.

## Agreement

I agree to the scope of work and terms outlined in this document. This plan may be amended with the consent of all parties named below.

| Name | Role |
| --- | --- |
| [insert name here] | Student |
| [insert name here] | Student |
| [insert name here] | Student |
| [insert name here] | Student |
| [insert name here] | Preceptor |
| [insert name here] | Faculty Adviser |
| [insert name here] | Teaching Assistant (TA) |
| [insert name here] | Instructor |
